# Case Report: Amiodarone-induced multi-organ toxicity

**DOI:** 10.3389/fcvm.2024.1401049

**Published:** 2024-07-17

**Authors:** Jingrui Yan, Yuanyuan Xu, Qiang Zhu

**Affiliations:** ^1^ Department of Gastroenterology, Shandong Provincial Hospital Affiliated to Shandong First Medical University, Jinan, Shandong, China; ^2^ Department of Medical Imaging, The Second Affiliated Hospital of Shandong First Medical University, Taian, Shandong, China

**Keywords:** amiodarone, atrial fibrillation, adverse reaction, thyroid, lung, liver, case report

## Abstract

**Background:**

Amiodarone is a class III antiarrhythmic drug that is commonly used in the clinic to treat ventricular arrhythmias and atrial fibrillation. We present a case report of the adverse effects of amiodarone and review its characteristics.

**Case report:**

A 73-year-old Asian female with a history of paroxysmal atrial fibrillation managed with amiodarone, well-controlled hypertension, and no substance abuse presented with gastrointestinal distress and dizziness, without chest pain or palpitations. Despite normal annual check-ups, she developed abnormal liver and thyroid function tests, and imaging revealed lung and liver changes suggestive of amiodarone toxicity. Discontinuation of amiodarone for sotalol led to symptom improvement and normalization of thyroid and liver functions, with imaging indicating recovery from interstitial fibrosis and reduced liver density.

**Discussion:**

Amiodarone, a widely used for treating ventricular and atrial arrhythmias, and with significant benefits in improving patient survival in cases of ventricular fibrillation. However, its long-term use is associated with serious adverse effects, including thyroid dysfunction, liver injury, and pulmonary toxicity, necessitating careful monitoring and management. Despite its efficacy, the need for research on early detection and management of amiodarone's side effects is crucial, highlighting the importance of regular monitoring and possibly adjusting therapy to mitigate these risks.

## Introduction

Amiodarone, classified as a Class III antiarrhythmic agent, is extensively prescribed in medical practices to address ventricular arrhythmias and atrial fibrillation. It has received approval from the U.S. Food and Drug Administration and is recommended by the European Society of Cardiology Guidelines for treating life-threatening ventricular arrhythmias (1). Amiodarone is an iodine-containing compound with high fat solubility. Amiodarone and its metabolite, desethylamiodarone, can accumulate in high concentrations in the thyroid, liver, lungs, and skin causing corresponding toxic reactions (2, 3). In this report, we detail a case of compromised hepatic, pulmonary, and thyroid functions due to chronic administration of Amiodarone.

## Case report

**Figure 1:**
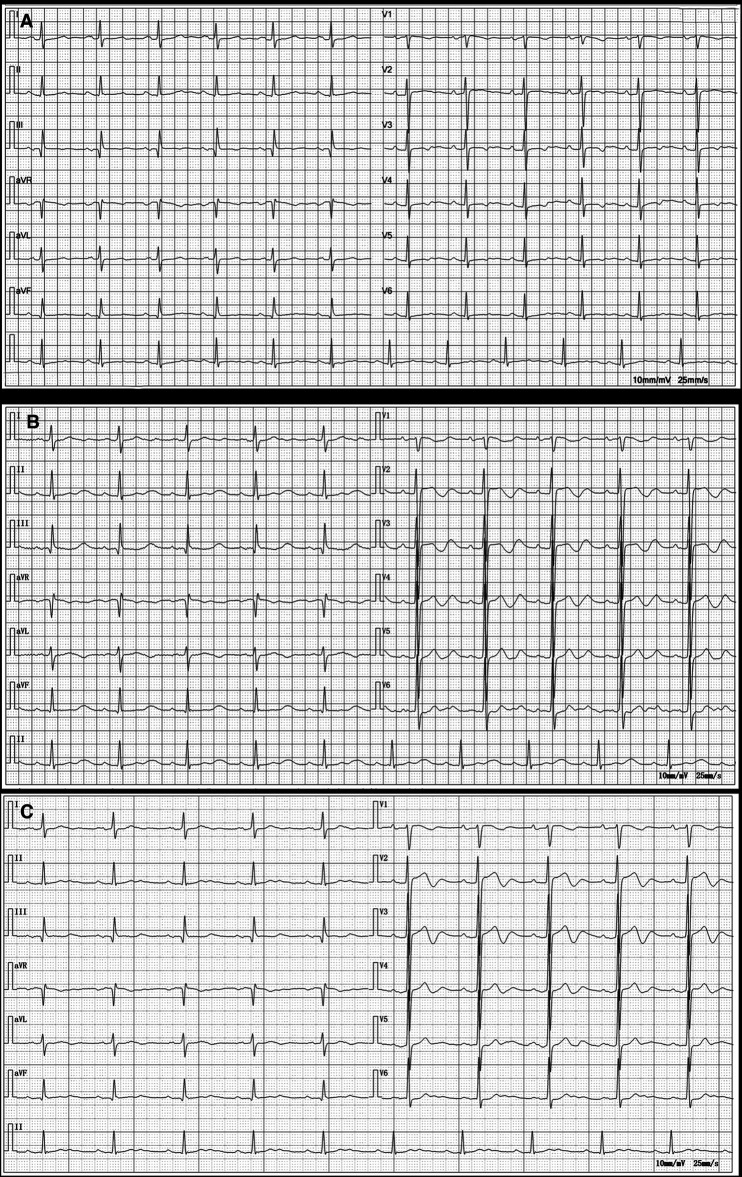
Electrocardiograms before amiodarone administration (2021.09.02), at the onset of organ damage (2023.08.13), and 1 week after discontinuation of the amiodarone (2023.08.21). (**A**) Electrocardiogram before amiodarone administration shows sinus rhythm with ST-T segment changes. (**B**) Electrocardiogram at the onset of organ damage shows sinus bradycardia; prolonged PR interval; widened P wave suggesting possible intra-atrial block; high voltage of left ventricular; increased U wave. (**C**) Electrocardiogram 1 week after discontinuation of the amiodarone shows sinus bradycardia; 1-degree atrioventricular block; T-wave change.

**Figure 2:**
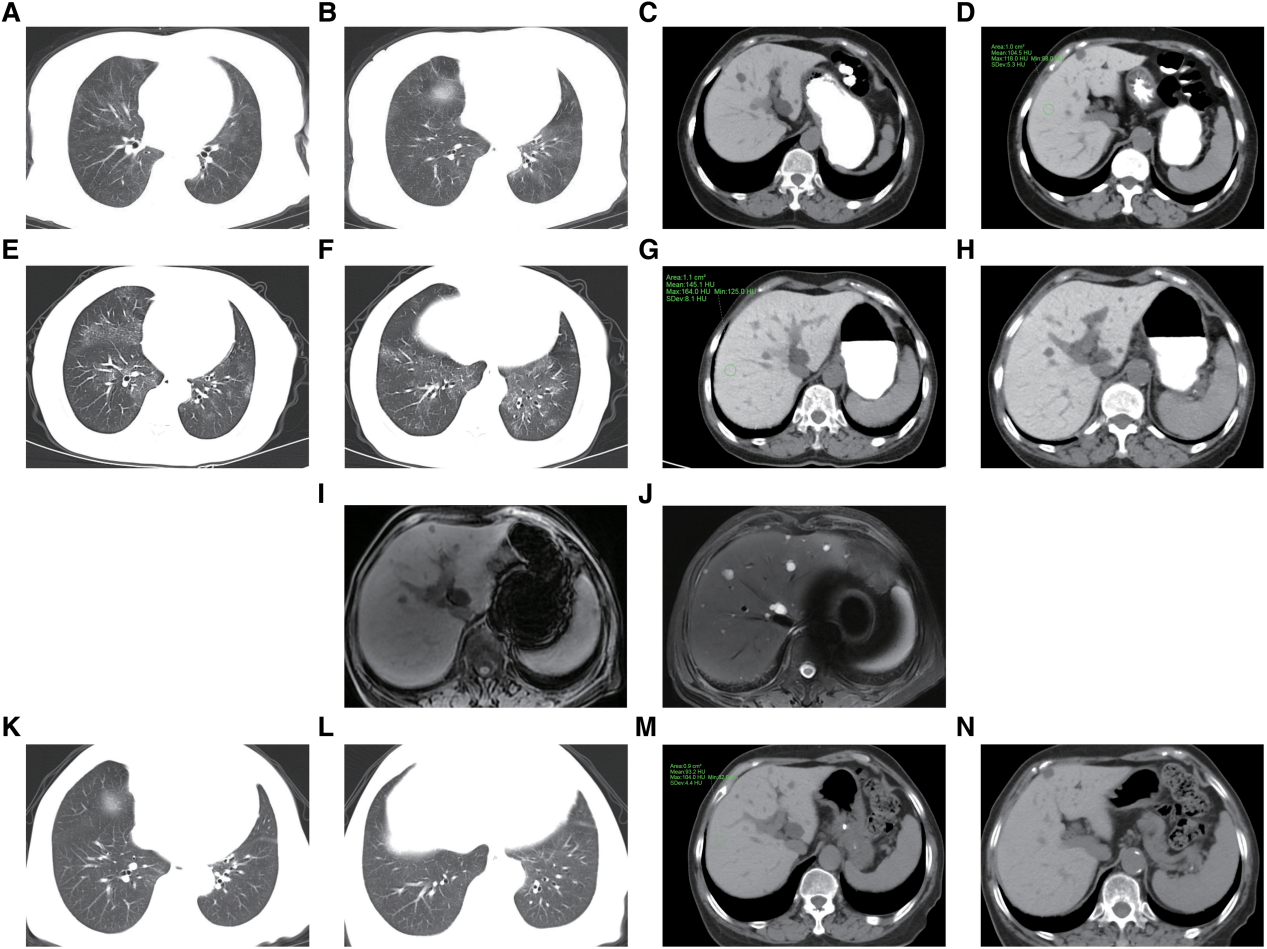
Imaging changes before (2022.02.14), at the onset of organ damage (2023.08.16), and 6 months (2024.02.09) after discontinuation of the amiodarone. (**A–D**) After 1 year (2022.02.14) of treatment with oral amiodarone (0.2 g/day), diffusely distributed ground-glass density shadows were seen in both lungs, with perifollicular hyperdense shadows, which were considered to be organizing pneumonia; the liver was hyperdense with a CT value of about 104 hu. (**E–J**) After 2 years (2023.08.16) of oral treatment with amiodarone, chest CT showed diffuse ground-glass shadows and fine reticular shadows in both lungs, with thickening of the interlobular septum, which was more significant than before; the density of the liver was markedly higher than before, with a CT value of about 145 HU. MRI of the liver showed the hepatic parenchyma was isointense on T1WI, and isointense on the fat-suppressed sequence of T2WI. (**K–N**) After stopping taking amiodarone for 6 months (2024.02.09), most of the lung lesions disappeared, and the density of the liver decreased, the CT value was about 93 HU.

**Figure 3:**
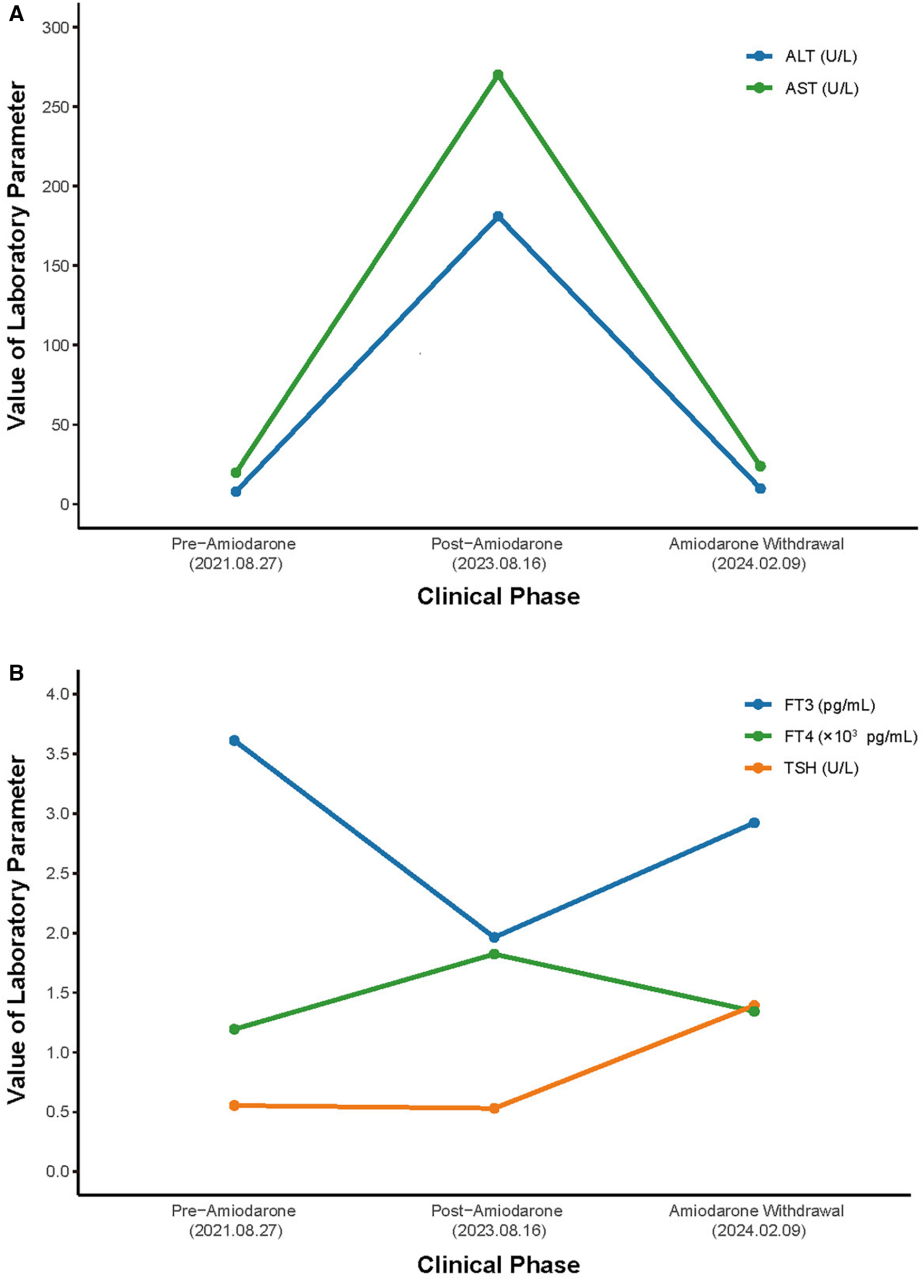
Hospital course of the patient. (**A**) Course of AST and ALT. (**B**) Course of TSH, FT3 and FT4. AST, aspartate aminotransferase; ALT, alanine aminotransferase; TSH, thyroid-stimulating hormone; FT4, free thyroxine; FT3, free triiodothyronine.

A 73-year-old Asian female presented to the hospital with a 2-day history of nausea, vomiting a small amount of mucus, bloating, belching, and occasional dizziness, but no acid reflux, heartburn, panic, palpitations, chest tightness, or pain. She claimed to weigh about 65 kg 6 months ago, which corresponded to a BMI of 26.04 kg/m^2^, which was in the overweight range. however, she had lost about 15 kg in the past 6 months. A decade ago, she was diagnosed with paroxysmal atrial fibrillation at our facility, initially managed with oral bisoprolol. She refused the catheter ablation at that point. Due to worsening palpitations two years prior, her treatment was adjusted to 0.2 g of amiodarone orally, twice daily. Since then, the patient has regularly took amiodarone as a drug to control atrial fibrillation. The patient was also being treated with Rosuvastatin calcium tablets, rivaroxaban, and amlodipine at the time of this admission. The patient has a 17-year history of well-controlled hypertension and no history of alcohol or injection drug use. Her vital signs were normal, except for mild hypertension (145/85 mmHg). Physical examination showed no abnormalities. Annual checkups (including thyroid function, liver function, and lung CT) before and following amiodarone initiation showed no significant findings until she developed the symptoms mentioned above, alongside abnormal liver (ALT 181 U/L, AST 270 U/L, ADA 25.6 U/L) and thyroid function tests (FT3: 1.96 pg/ml, FT4: 1.82 pg/ml). (Table 1) The electrocardiogram demonstrated sinus bradycardia, prolonged PR interval, P wave widening suggestive of intra-atrial block, high voltage of left ventricular, and increased U wave amplitude (Figure 1). The patient exhibited mild hypokalemia, which rapidly improved with oral potassium supplementation. Cardiac ultrasound indicated left atrial enlargement, degenerative aortic valve with mild regurgitation, mild tricuspid regurgitation, and left ventricular diastolic dysfunction. Chest CT scanning showed diffuse ground-glass density shadow and fine reticular shadow in both lungs, and thickening of interlobular septum; abdominal CT scanning showed diffuse increase in liver density, with a CT value of 125–164 HU, which was considered to be iron overload, but further MRI scanning and enhancement examination of liver showed that the liver parenchymal signal did not show abnormally low on T2-weighted imaging, which was excluded (Figure 2). Liver and lung injuries due to amiodarone were considered in conjunction with the history and laboratory tests. Retrospective lung CT analysis from an annual check-up suggested amiodarone-associated pulmonary mechanized pneumonitis and liver density increase (Figure 2). After discontinuing amiodarone for sotalol, her symptoms improved, and follow-up tests 6 months later showed normalized thyroid and liver functions (Figure 3), with lung CT indicating interstitial fibrosis absorption and reduced liver density (Figure 2). Therefore, invasive procedures such as liver, lung and thyroid biopsies were not performed.

**Table 1:** Changes in laboratory indices before, after, and following discontinuation of amiodarone therapy.

Laboratory test (Reference range)	Pre-amiodarone therapy (2021.08.27)	Post-amiodarone therapy (2023.08.16)	Amiodarone withdrawal (2024.02.09)
TSH (0.55–4.78 U/L)	0.551	0.527	1.389
FT3 (2.3–4.2 pg/ml)	3.61	1.96	2.92
TPOAb (0–60 IU/ml)	41.7	0.38	0.365
TgAb (0–60 IU/ml)	<15.0	14.5	2.95
FT4 (0.89–1.76 × 10^3^ pg/ml)	1.19	1.82	1.34
ALT (7–45 U/L)	8	181	10
AST (13–35 U/L)	20	270	24
AST/ALT ratio	2.5	1.49	2.4
GGT (7–45 U/L)	12	45	17
ALP (40–150 U/L)	67	104	93

TSH, thyroid-stimulating hormone; FT3, free triiodothyronine; TPOAb, anti-thyroid peroxidase antibodies; TgAb, anti-thyroglobulin antibodies; FT4, free thyroxine; ALT, alanine aminotransferase; AST, aspartate aminotransferase; GGT, gamma-glutamyl transferase; ALP, alkaline phosphatase.

## Discussion

Amiodarone recognized as a class III antiarrhythmic agent (4), operates by blocking potassium efflux in delayed rectifier channels, coupled with the non-competitive inhibition of beta receptors and suppression of slow calcium channels (5). Thus, amiodarone, as a classical antiarrhythmic drug, has both anti-ventricular and anti-atrial arrhythmic effects (6). In patients with atrial fibrillation, amiodarone may be used for resuscitation in patients with no hemodynamic abnormalities and who are symptomatic, especially if electrical cardioversion is unsuitable. It achieves acute atrial fibrillation reversal in 35%–65% of patients (7) and is also beneficial in sustaining sinus rhythm post-electrical resuscitation (1). Although the latest guidelines (8) state that catheter ablation is a key therapeutic intervention primarily used for rhythm control in patients who have symptomatic atrial fibrillation, particularly when drug therapy is ineffective, not tolerated, or not preferred. However, there are specific contraindications to performing catheter ablation, which include: the presence of active infection, uncontrolled heart failure, severe pulmonary hypertension, severe left atrial enlargement, blood clots in the heart, and uncontrolled bleeding or high risk for bleeding. Additionally, the guidelines indicate that clinical trials have shown significant benefits of catheter ablation in individuals under the age of 70, but the clinical efficacy in those over the age of 70 remains uncertain. Moreover, the guidelines recommend the use of Amiodarone or Dronedarone as anti-arrhythmic drugs for patients with hypertension or left ventricular hypertrophy accompanied by atrial fibrillation, and the use of beta-blockers or calcium channel blockers as rate control drugs. In this case, the patient presented with atrial fibrillation but the cardiac ultrasound suggested left atrial enlargement, besides, she is 73 years old with a history of hypertension for more than 17 years, thus making catheter ablation an unselected option, her symptoms did not improve after first-line treatment, and she took amiodarone to effectively control palpitations but developed new conditions such as impairment of thyroid function, hepatic function, and pulmonary function. Given amiodarone's extensive half-life of 50–60 days, its therapeutic effects remain viable for up to 3 months after discontinuation (9), as evidenced by the significant improvement in the patient's lung and liver health 6 months following drug cessation.

The most common adverse effects caused by amiodarone are nausea, vomiting and abnormal taste, and the most serious adverse effects are liver damage and lung damage. Moreover, owing to its structural similarity to thyroid hormones (10), amiodarone disrupts thyroid hormone synthesis, leading to Amiodarone-induced hypothyroidism (AIH) in iodine-sufficient populations. This condition is characterized by elevated T4 levels, reduced T3, and slightly increased TSH (11). Research (12) indicates that within one year of amiodarone treatment, approximately 5% of patients experience some form of thyroid dysfunction. The presence of both female and TPO antibodies, the two main risk factors, implies a 13.5-fold higher relative risk of developing AIH (13). Clinically, amiodarone is prescribed at dosages ranging from 100 to 600 mg/day, translating to 3–21 mg of iodine, far exceeding the daily recommended iodine intake of 150 µg (14). The absence of routine amiodarone serum concentration assessments hinders prompt diagnosis and management of its toxic effects. Typically, AIH resolves 2–4 months after discontinuation of the drug (15).

Amiodarone pulmonary toxicity is usually manifested as interstitial pneumonia and hypersensitivity syndrome (16), though cases of ARDS and the emergence of pulmonary nodules have also been documented (17, 18). The morbidity rate ranges from 0.5% to 17% (19), with a lethality rate of 10% to 33%, varying with the patient's overall health (20). Patients most commonly present with dyspnea, followed by cough, fever, nausea, and malaise (21). The underlying pathogenesis involves cytotoxicity towards type 2 alveolar epithelial cells, activation of the angiotensin system, and genetic predispositions (22). Pulmonary function tests often show reduced forced vital capacity (FVC) and a marginal decrease in the diffusing capacity for carbon monoxide (DLCO). Chest CT scans typically reveal patchy or diffuse ground-glass opacities (23), while microscopic analysis may display foamy alveolar macrophages and cytoplasmic lamellar bodies (24), indicative of amiodarone pulmonary toxicity. Moreover, serum Krebs von den Lungen-6 (KL-6) is also a reliable biomarker for the management of interstitial lung diseases. Study shows KL-6 is elevated in 70%–100% of patients with interstitial lung diseases induced by various reasons (25). Risk factors include the dosage and duration of amiodarone treatment, patient age, and medical history. Therefore, amiodarone should be used with caution in patients with a previous history of pulmonary disease (e.g., asthma, COPD) to avoid its pulmonary side effects. A recent study (26) included 6,039 amiodarone-exposed patients and an equal number of matched controls and concluded that in contemporary AF patients, low-dose amiodarone was associated with a trend towards increased risk of interstitial lung disease by 15%–45%, a clinically negligible change in absolute risk (maximum of 1.8%), no increased risk of primary lung cancer, and a lower risk of all-cause mortality. In this case, the patient's respiratory symptoms were not obvious, no pulmonary function tests or biopsies were performed, and only multiple patchy ground-glass shadows were found in the chest CT at the annual health checkup. Without targeted treatment for pulmonary toxicity, significant improvement in the patient's chest CT images was noted 6 months post-amiodarone discontinuation.

There are fewer studies related to the hepatotoxicity of amiodarone, which manifests itself as minor liver injury such as elevated aminotransferases (27). However, instances of fatal hepatic failure have been reported. It is generally recommended to reduce or discontinue amiodarone when AST or ALT levels exceed twice the upper limit of normal. One study observed that approximately one-quarter of patients exhibited asymptomatic elevated serum aminotransferases, whereas symptomatic hepatitis manifestations were noted in only 3% of cases (28). The etiology of amiodarone-induced liver injury remains uncertain, with theories suggesting direct hepatocyte membrane damage by the solvent polysorbate 80 during intravenous administration (29) and mitochondrial damage due to the accumulation of lipid-rich substances in lysosomes (30). Furthermore, the patient in this case was also receiving rosuvastatin calcium treatment. Amiodarone impacts statin metabolism by inhibiting the mitochondrial enzyme CYP3A4, which is a potential cause of hepatic injury (31). The primary treatment for amiodarone-induced liver injury is drug cessation, typically followed by supportive therapy. In cases of severe hepatic failure, liver transplantation may be considered (32). Monitoring serum aminotransferases, bilirubin, and blood ammonia levels is essential for assessing treatment efficacy and preventing complications like hepatic encephalopathy. In this case, the patient presented with a rise in AST of about eight times the normal value, a fourfold rise in ALT, and symptoms such as nausea and abdominal distension, which were seen to improve significantly after discontinuation of the drug.

Despite its widespread use in treating arrhythmic conditions, amiodarone's side effects have been underemphasized, with a lack of large-scale clinical investigations to track its adverse reactions. This oversight underscores the urgent need for research focused on early detection methods, presenting a wide scope for clinical relevance and research opportunities. The case of amiodarone-induced multiorgan damage with early manifestation of gastrointestinal symptoms serves as a reminder for clinicians. When patients with atrial fibrillation receiving amiodarone develop gastrointestinal symptoms, respiratory symptoms, or thyroid function abnormalities, amiodarone-induced multiorgan toxicity should be considered promptly, and the medication should be promptly discontinued, or alternative therapeutic options should be selected. However, this case report has limitations. First, due to the technical limitations of the hospital, it is a pity not to measure amiodarone blood concentration and KL-6 to assess pulmonary fibrosis. Second, the patient did not present with respiratory system-related clinical symptoms, which led to the lack of appropriate treatment for lung injury. Third, the long-term follow-up period for the patient was insufficient; therefore, we will continue to contact the patient regularly. In summary, clinicians must standardize amiodarone administration, adjust the dose of medication according to the patient's BMI, and ensure regular monitoring of liver function, blood biochemistry, and additional laboratory metrics. Furthermore, assessing serum drug levels periodically could enhance therapeutic efficacy and reduce adverse effects.

## Conclusion

Our case represents an elderly female patient who experienced thyroid, hepatic, and pulmonary impairments following prolonged low-dose oral administration of amiodarone. Given amiodarone's unique pharmacokinetic properties, organ damage persisted even after cessation of the drug, necessitating prompt dosage adjustments to mitigate its toxic effects. It underscores the importance of timely follow-up assessments by clinicians to facilitate the early identification and effective management of adverse reactions, thereby averting more severe outcomes.

## Data Availability

The raw data supporting the conclusions of this article will be made available by the authors, without undue reservation.
